# The Facial Expressive Action Stimulus Test. A test battery for the assessment of face memory, face and object perception, configuration processing, and facial expression recognition

**DOI:** 10.3389/fpsyg.2015.01609

**Published:** 2015-10-29

**Authors:** Beatrice de Gelder, Elisabeth M. J. Huis in ‘t Veld, Jan Van den Stock

**Affiliations:** ^1^Department of Cognitive Neuroscience, Maastricht UniversityMaastricht, Netherlands; ^2^Department of Psychiatry and Mental Health, University of Cape TownCape Town, South Africa; ^3^Department of Medical and Clinical Psychology, Tilburg UniversityTilburg, Netherlands; ^4^Laboratory for Translational Neuropsychiatry, Department of Neurosciences, KU LeuvenLeuven, Belgium; ^5^Old Age Psychiatry, University Hospitals LeuvenLeuven, Belgium

**Keywords:** face recognition, face memory, emotion recognition, configural face processing, inversion effect, experimental task battery

## Abstract

There are many ways to assess face perception skills. In this study, we describe a novel task battery FEAST (Facial Expressive Action Stimulus Test) developed to test recognition of identity and expressions of human faces as well as stimulus control categories. The FEAST consists of a neutral and emotional face memory task, a face and shoe identity matching task, a face and house part-to-whole matching task, and a human and animal facial expression matching task. The identity and part-to-whole matching tasks contain both upright and inverted conditions. The results provide reference data of a healthy sample of controls in two age groups for future users of the FEAST.

## Introduction

Face recognition is one of the most ubiquitous skills. The neural underpinnings of face perception are still a matter of debate. This is not surprising when one realizes that a face has a broad range of attributes. Identity is but one of these, and it is not clearly understood yet how a deficit in that area affects perception and recognition of other aspects of face perception. Prosopagnosia or absence of normal face identity recognition is one of the most peculiar neuropsychological symptoms and it has shed some light on the nature of face perception (de Gelder and Van den Stock, 2015). The term referred originally to loss of face recognition ability in adulthood following brain damage (Bodamer, 1947). Prosopagnosia can have a profound impact on social life, as in extreme cases the patients have difficulty recognizing the face of their spouse or child. More recently it has also been associated with neurodegenerative syndromes like fronto-temporal lobe degeneration (FTLD) (Snowden et al., 1989) and neurodevelopmental syndromes like cerebellar hypoplasia (Van den Stock et al., 2012b). In addition to the acquired variant, there is now general consensus on the existence of a developmental form, i.e., developmental prosopagnosia (DP). A recent prevalence study reported an estimate of 2.5% (Kennerknecht et al., 2006) and indicates that DP typically shows a hereditary profile with an autosomal dominant pattern.

In view of the rich information carried by the face, an assessment of specific face processing skills is crucial. Two questions are central. One, what specific dimension of facial information are we focusing on, and two, is its loss specific for faces. To date, there is no consensus or golden standard regarding the best tool and performance level that allows diagnosing individuals with face recognition complaints as “prosopagnosic.” Several tests and tasks have been developed, such as the Cambridge Face Memory Test (Duchaine and Nakayama, 2006), the Benton Facial Recognition Test (Benton et al., 1983), the Cambridge Face Perception Task (Dingle et al., 2005), the Warrington Recognition Memory Test (Warrington, 1984) and various tests using famous faces (such as adaptations of the Bielefelder famous faces test, Fast et al., 2008). These each provide a measure or a set of measures relating to particular face processing abilities, e.g., matching facial identities or rely on memory for facial identities which is exactly what is problematic in people with face recognition disorders. More generally, beyond the difference between perception and memory, there is not yet a clear understanding of how the different aspects of normal face perception are related. So testing of face skills should cast the net rather wide. A test battery suitable for the assessment of prosopagnosia should take some additional important factors into account. Firstly, to assess the face specificity of the complaints, the test battery should include not only tasks with faces, but also an equally demanding condition with control stimuli that are visually complex. Secondly, an important finding classically advanced to argue for a specialization for faces regards the configural way in which we seem to process faces, so the task should enable the measurement of configural processing of faces and objects. The matter of configuration perception also has been tackled in several different ways, such as with the composite face task (Young et al., 1987), the whole-part face superiority effect (Tanaka and Farah, 1993) or more recently, using gaze-contingency (Van Belle et al., 2011). We choose to focus on the classical face inversion effect (Yin, 1969; Farah et al., 1995), whose simple method lends itself very well to study object inversion effects. Next, besides using the inversion effect, configuration- vs. feature-based processing can also be investigated more directly by part-to-whole matching tasks (de Gelder et al., 2003). Furthermore, previous studies have found positive relationships between the ability to process faces configurally and face memory (Richler et al., 2011; Huis in ‘t Veld et al., 2012; Wang et al., 2012; DeGutis et al., 2013) indicating that configural processing might facilitate memory for faces.

Additionally, there is accumulating evidence in support of an interaction between face identity and face emotion processing (Van den Stock et al., 2008; Chen et al., 2011; Van den Stock and de Gelder, 2012, 2014) and there is increasing evidence that configuration processing is positively related to emotion recognition ability (Bartlett and Searcy, 1993; Mckelvie, 1995; Calder et al., 2000; White, 2000; Calder and Jansen, 2005; Durand et al., 2007; Palermo et al., 2011; Tanaka et al., 2012; Calvo and Beltrán, 2014). We therefore extended our test battery with tasks targeting emotion recognition and emotion effects on face memory, by adding an emotional face memory task and a facial expression matching task. To stay with the rationale of our test that each skill tested with faces must also be tested with a selected category of control objects, we used canine face expressions.

Taking all these aspects into account, we constructed a face perception test battery labeled the Facial Expressive Action Stimulus Test (FEAST). The FEAST is designed to provide a detailed assessment of multiple aspects of face recognition ability. Most of the subtests have been extensively described and validated on the occasion of prosopagnosia case reports and small group studies (de Gelder et al., 1998, 2000, 2003; de Gelder and Rouw, 2000a,b,c, 2001; Hadjikhani and de Gelder, 2002; de Gelder and Stekelenburg, 2005; Righart and de Gelder, 2007; Van den Stock et al., 2008, 2012a, 2013; Huis in ‘t Veld et al., 2012). But so far the test battery was not presented systematically as it had not been tested on a large sample of participants receiving the full set of subtests. Here, we report a new set of normative data for the finalized version of the FEAST, analyze the underlying relationships of the tasks, and freely provide the data and stimulus set to the research community for scientific purposes.

## Materials and methods

### Subjects

The participants were recruited between 2012 and 2015 from acquaintances of lab members and research students. Participation was voluntarily and no monetary reward was offered. The following inclusion criteria were applied: right-handed, minimally 18 years old, normal or corrected-to-normal vision and normal basic visual functions as assessed by the Birmingham Object Recognition Battery (line length, size, orientation, gap, minimal feature match, foreshortened view, and object decision) (Riddoch and Humphreys, 1992). A history of psychiatric or neurological problems, as well as any other medical condition or medication use which would affect performance, or history of a concussion, were exclusion criteria. This study was carried out in accordance with the recommendations and guidelines of the Maastricht University ethics committee, the “Ethische Commissie Psychologie” (ECP). The protocol was approved by the Maastricht University ethics committee (ECP-number: ECP-128 12_05_2013).

In total, 61 people participated in the study. Three subjects were 80, 81, and 82 years old. Even though they adhered to every inclusion criteria, they were excluded from the analyses due to being outliers on age (more than 2 standard deviations from the mean). The sample thus consisted of 58 participants, between 18 and 62 years old (*M* = 38, *SD* = 15). Of those, 26 are male, between 19 and 60 years old (*M* = 38, *SD* = 15) and 32 women between 18 and 62 years old (*M* = 39, *SD* = 16). There are no differences in age between the genders [*t*_(1, 56)_ = −0.474, *p* = 0.638].

**Figure 1:**
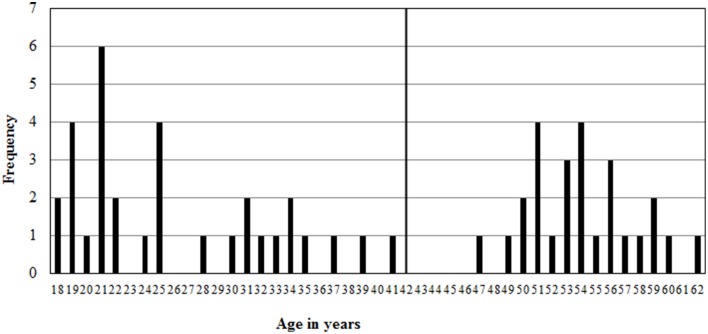
**Age distribution of the sample with the young adult group between 18 and 41 years old, and a middle aged group between 47 and 62 years old**.

However, an age distribution plot (see Figure 1) reveals a gap, where there are only 6 participants between 35 and 49. Therefore, the sample is split in two: one “young adult” group, younger than 42 and a “middle aged” group of participants between 47 and 62 years old. The young adult age group consisted of 15 men between 19 and 37 years old, (*M* = 26, *SD* = 6) and 17 women between 18 and 41 years old (*M* = 26, *SD* = 8). The middle aged group consisted of 11 men between 47 and 60 years old (*M* = 53, *SD* = 4) and 15 women between 50 and 62 years old (*M* = 55, *SD* = 3).

### Experimental stimuli and design

The face and shoe identity matching task, face and house part-to-whole matching task, Neutral and Emotion Face Memory task (FaMe-N and FaMe-E) have been previously described including figures of stimulus examples (Huis in ‘t Veld et al., 2012).

#### Face and shoe identity matching task and the inversion effect

**Figure 2:**
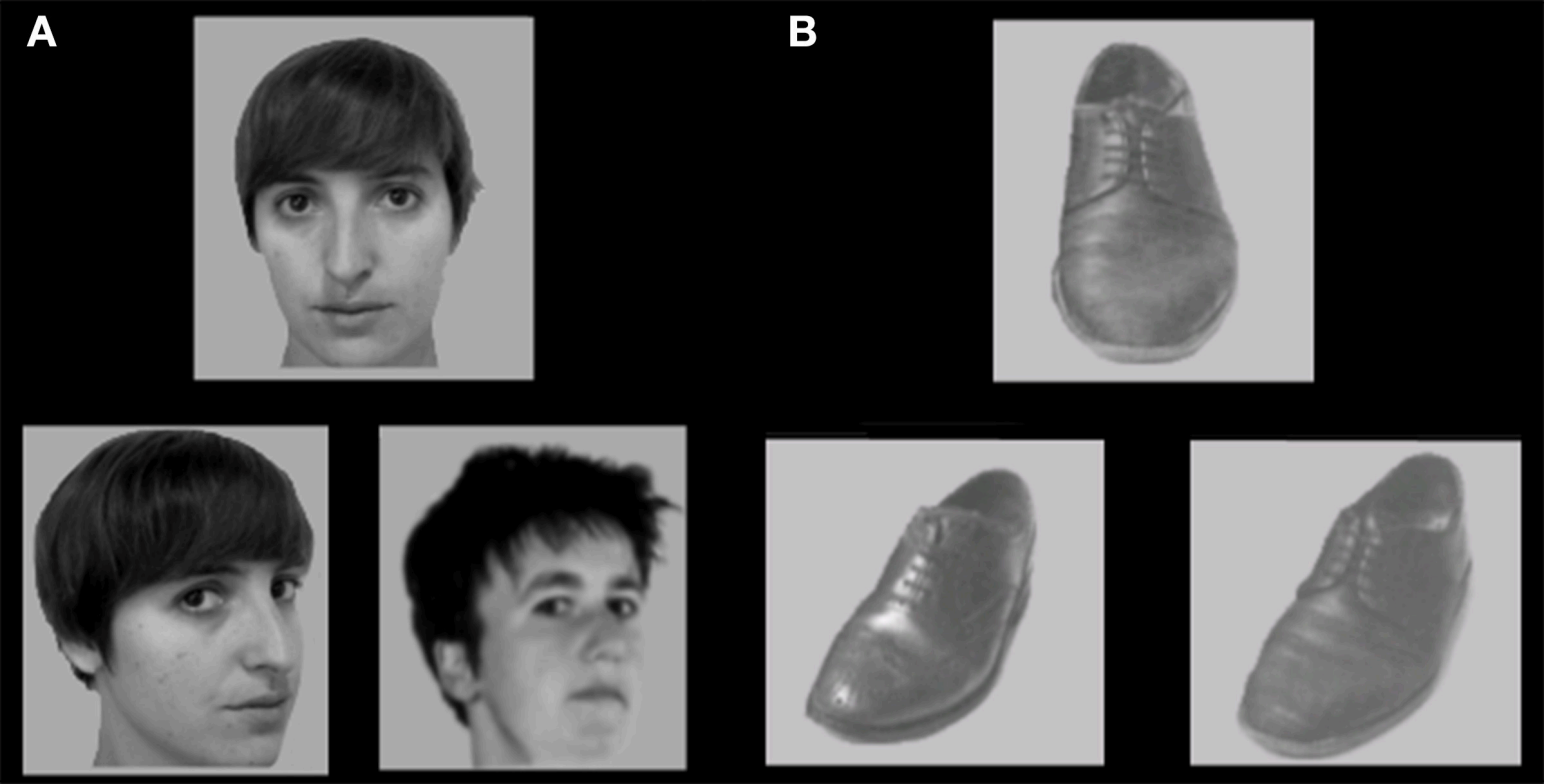
**Stimulus example of (A) upright faces and (B) upright shoes in the face and shoe identity matching task**. Some identities are different from the actual stimuli due to copyright and permissions.

The face and shoe identity-matching task (de Gelder et al., 1998; de Gelder and Bertelson, 2009) was used to assess identity recognition and the inversion effect for faces and objects. The test contained 4 conditions with a 2 category (faces and shoes) × 2 orientation (upright and inverted) factorial design. The materials consisted of greyscale photographs of shoes (8 unique shoes) and faces (4 male, 4 female; neutral facial expression) with frontal view and ¾ profile view. A stimulus contained three pictures: one frontal view picture on top and two ¾ profile view pictures underneath. One of the two bottom pictures (target) was of the same identity as the one on top (sample) and the other was a distracter. The target and distracter pictures of the faces were matched for gender and hairstyle. Each stimulus was presented for 750 ms and participants were instructed to indicate by a button press which of the two bottom pictures represented the same exemplar as the one on top. Participants were instructed to answer as quickly but also as accurately as possible, and responses during stimulus presentation were collected. Following the response, a black screen with a fixation cross was shown for a variable duration (800–1300 ms). The experiment consisted of four blocks (one block per condition). In each block, 16 stimuli were presented 4 times in a randomized order, adding up to a total of 64 trials per block. Each block was preceded by 4 practice trials, during which the participants received feedback about their performance (see Figure 2).

#### Face and house part-to-whole matching task

**Figure 3:**
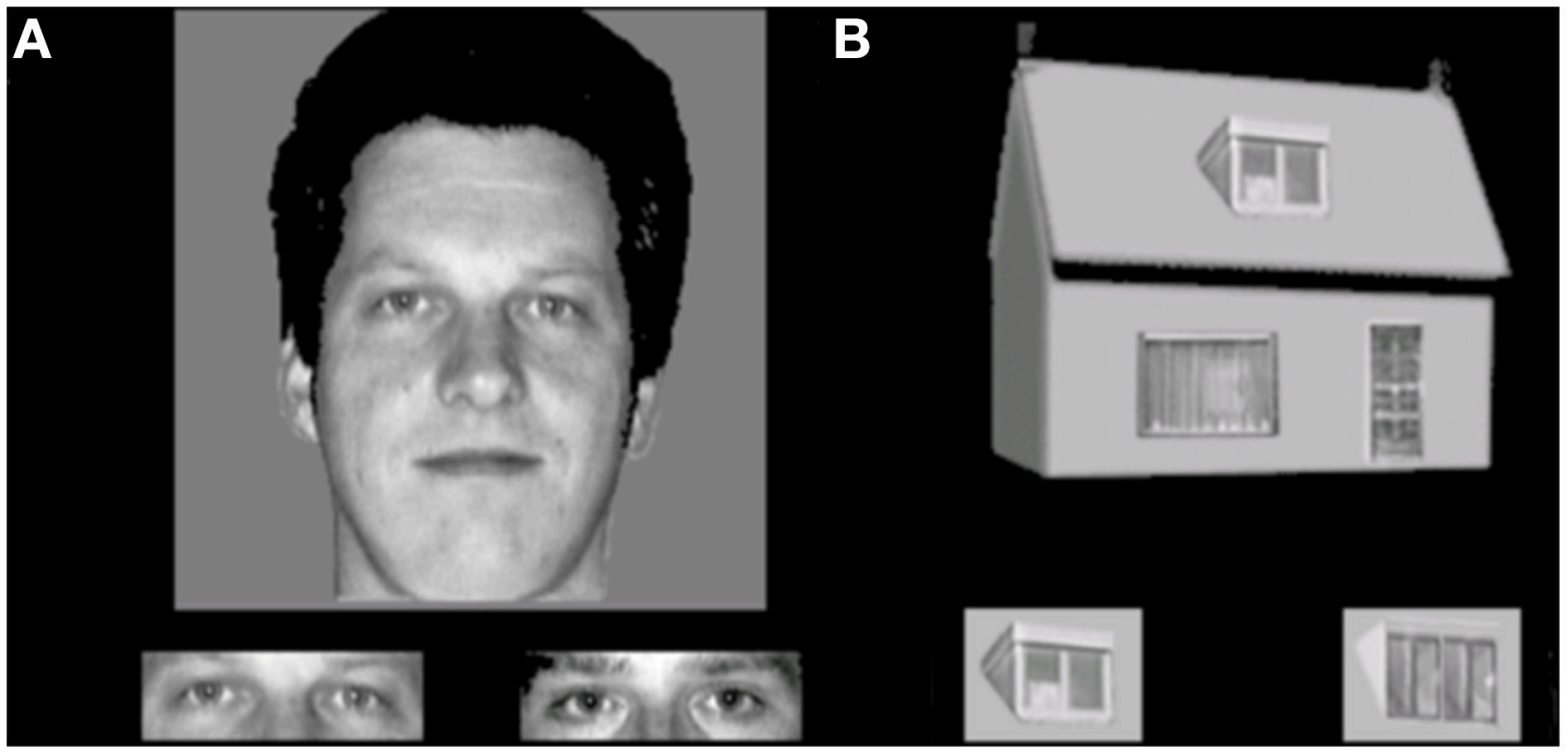
**Stimulus examples of an (A) upright face and eyes and (B) upright house and windows trial in the face and house part-to-whole matching task**.

This task is developed to assess holistic processing. The test also consisted of 4 conditions, with a 2 category (faces and houses) × 2 orientation (upright and inverted) factorial design. Materials consisted of grayscale pictures of eight faces (four male; neutral facial expression, photographed in front view and with direct gaze) and eight houses. From each face, part-stimuli were constructed by extracting the rectangle containing the eyes and the rectangle containing the mouth. House-part stimuli were created using a similar procedure, but the parts consisted of the door or window. The trial procedure was similar to the face and object identity matching task, where a whole face or house was presented on top (sample), with a target part-picture and a distractor part-picture presented underneath. Each trial was presented for 750 ms and participants were instructed to indicate by a button press which of the two bottom pictures represented the same exemplar as the one on top. Participants were instructed to answer as quickly but also as accurately as possible, and responses during stimulus presentation were collected. Following the response, a black screen with a fixation cross was shown for a variable duration (800–1300 ms). The experiment consisted of eight blocks (two blocks per condition). In each block, 16 stimuli were presented 2 times in a randomized order, adding up to a total of 32 trials per block and 64 trials per condition. Within blocks, the presentation of the two parts (eyes or mouth, window or door) was randomized in order to prevent participants to pay attention only to one specific feature. The first block of each condition was preceded by 4 practice trials, during which the participants received feedback about their performance (see Figure 3).

#### Facial expression matching task (FEM-H and FEM-C)

The FEM is a match-to-sample task used to measure emotion recognition ability in both human and canine faces. The experiment was divided into two parts. The first part consisted of human facial expressions (anger, fear, happy, sad, surprise, disgust). The materials consisted of grayscale photographs of facial expressions of 34 female identities and 35 male identities taken from the Karolinska Directed Emotional Faces (KDEF) (Lundqvist et al., 1998). This task has been used previously in Van den Stock et al. (2015). A stimulus consisted of three pictures: one picture on top (sample) and two pictures underneath. One of the two bottom pictures showed a face expressing the same emotion as the sample, the other was a distracter. The target and distracter pictures of the faces were matched for gender for the human stimuli. Each trial was presented until a response was given, but participants were instructed to answer as quickly and accurately as possible. Following the response, a black screen with a fixation cross was shown for a variable duration (800–1300 ms). Each emotional condition contained 10 trials (5 male) in which the target emotion was paired with a distracter from each of the other emotions once per gender, resulting in 60 trials in total. The first part was preceded by 4 practice trials, during which the participants received feedback about their performance.

**Figure 4:**
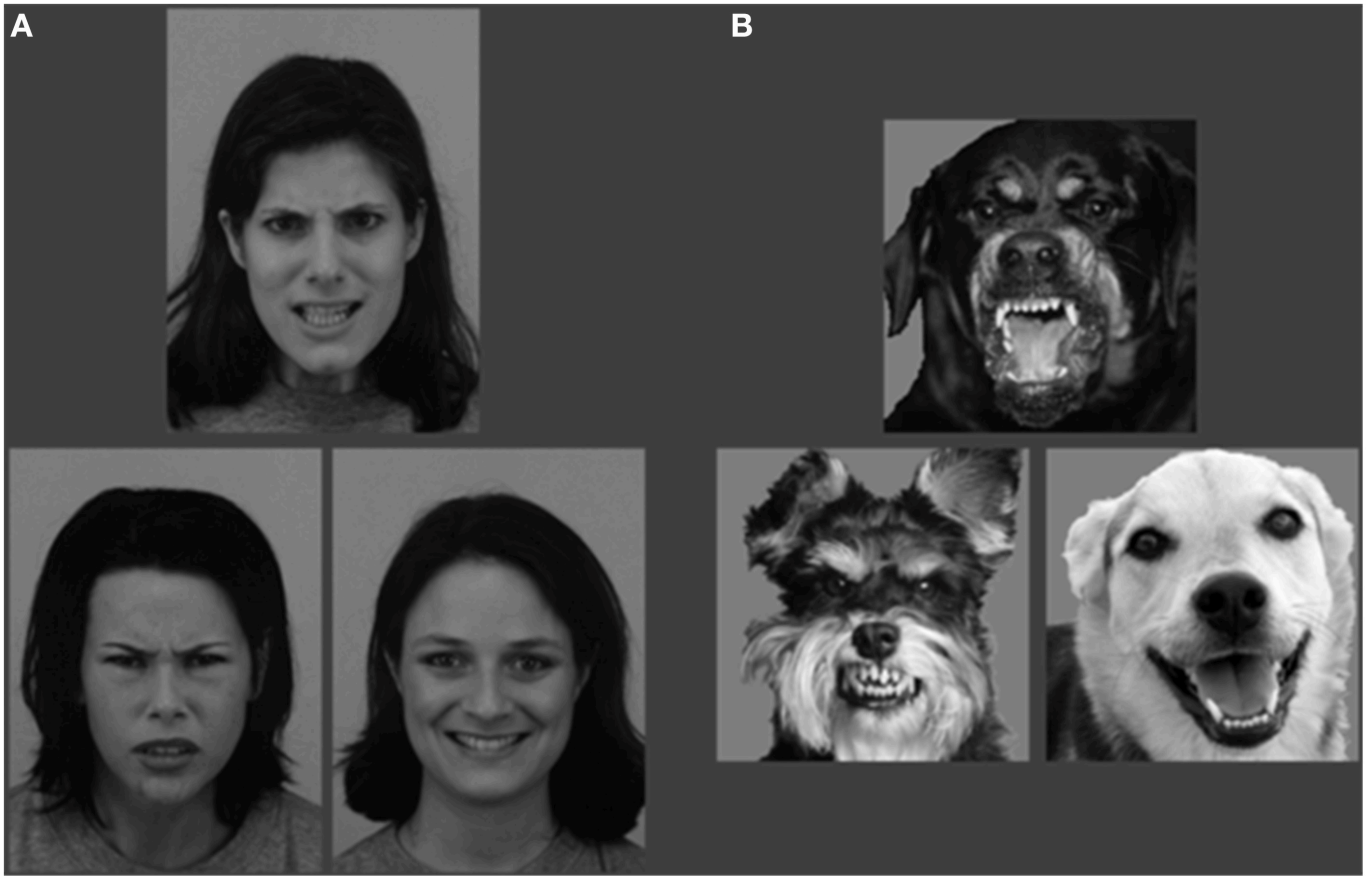
**Example stimulus of the Facial Expression Matching Task with an angry target and happy distracter stimulus trial for the (A) human and (B) canine experiment**.

The second part consisted of canine facial expressions. In total, 114 pictures of dogs which could be perceived as angry (17), fearful (27), happy (17), neutral (29), and sad (24) were taken from the internet by EH. These pictures were validated in a pilot study using 28 students of Tilburg University in exchange for course credit. The participants indicated of each photo whether they thought the dog was expressing anger, fear, happiness, sadness or no emotion in particular (neutral) and secondly, how intense they rated the emotional expression on a scale from one to five. Twelve angry, twelve fearful, and twelve happy canine expressions were accurately recognized by more than 80% of the participants and used in the experiment. The canine part consisted of 72 trials in total, 24 per emotion condition, in which each target emotion was paired with each of the distracter emotions 12 times. The experiment was preceded by 2 practice trials, during which the participants received feedback about their performance (see Figure 4).

#### Neutral face memory task (FaMe-N)

Based on the Recognition Memory Test (Warrington, 1984), the FaMe-N consists of an encoding and a recognition phase. The stimuli consist of 100 grayscale Caucasian faces (50 male) with a neutral facial expression, in front view, with frontal eye gaze. The stimuli were taken from a database created at Tilburg University. Trials in the encoding phase consisted of the presentation of a single stimulus for 3000 ms, followed by a black screen with a white fixation cross with a duration of 1000 ms. Participants were instructed to encode each face carefully and told that their memory for the faces would be tested afterwards. The encoding block consisted of 50 trials.

**Figure 5:**
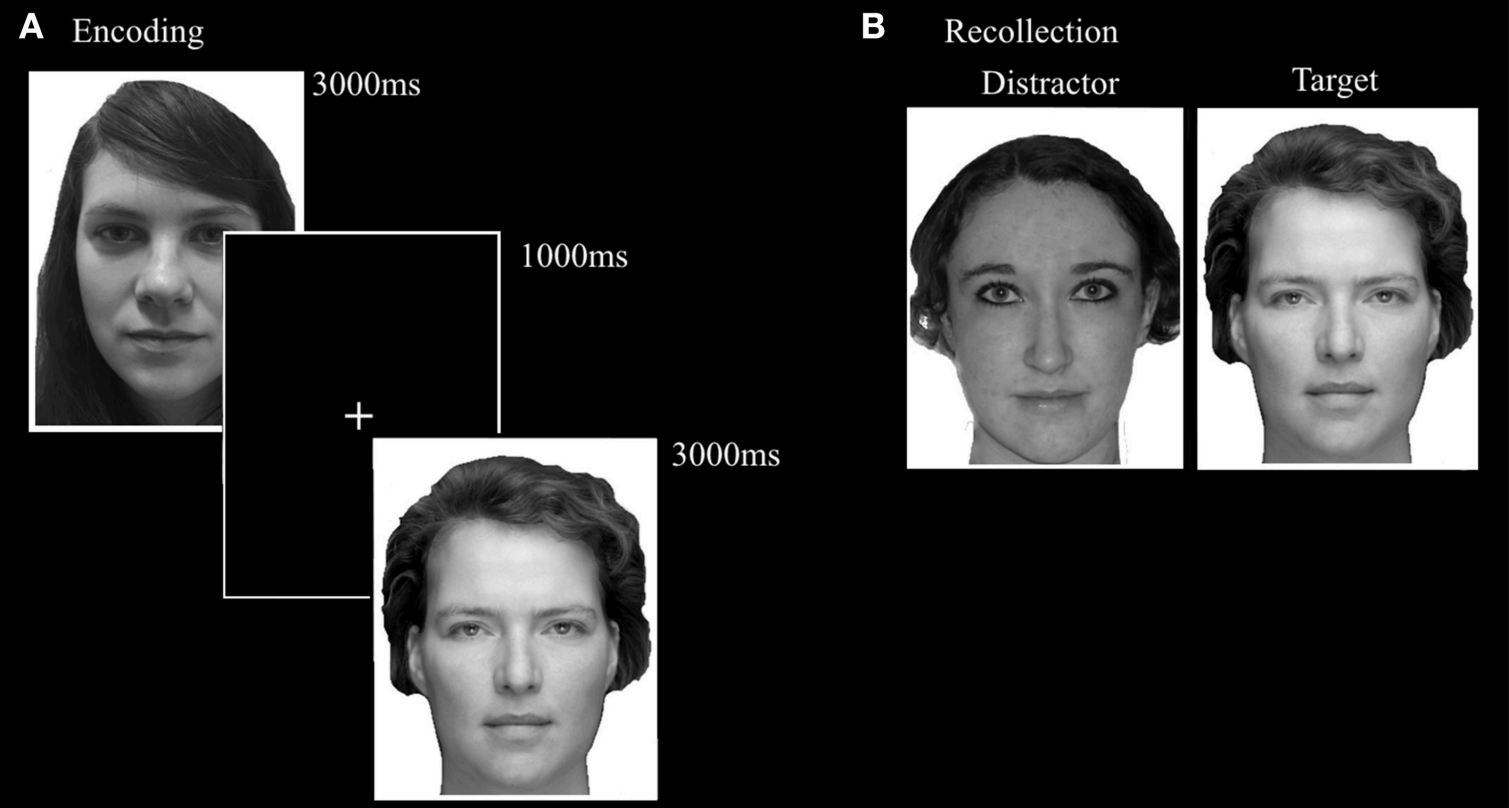
**Trial setup examples of the (A) encoding phase and (B) recollection phase of the FaMe-N**. Identities are different from the actual stimuli due to copyright and permissions.

The recognition phase immediately followed upon the encoding phase. A trial in the recognition phase consisted of the simultaneous presentation of two adjacent faces. One was the target face and was also presented in the encoding phase. The other face was not previously presented in the encoding phase and served as distracter. Fifty trials were randomly presented and target and distractor presentation side were evenly distributed. Participants were instructed to indicate as quickly and also as accurately as possible which face was also presented in the encoding phase. The stimulus pairs were matched for gender and hairstyle (see Figure 5).

#### Emotional face memory task (FaMe-E)

**Figure 6:**
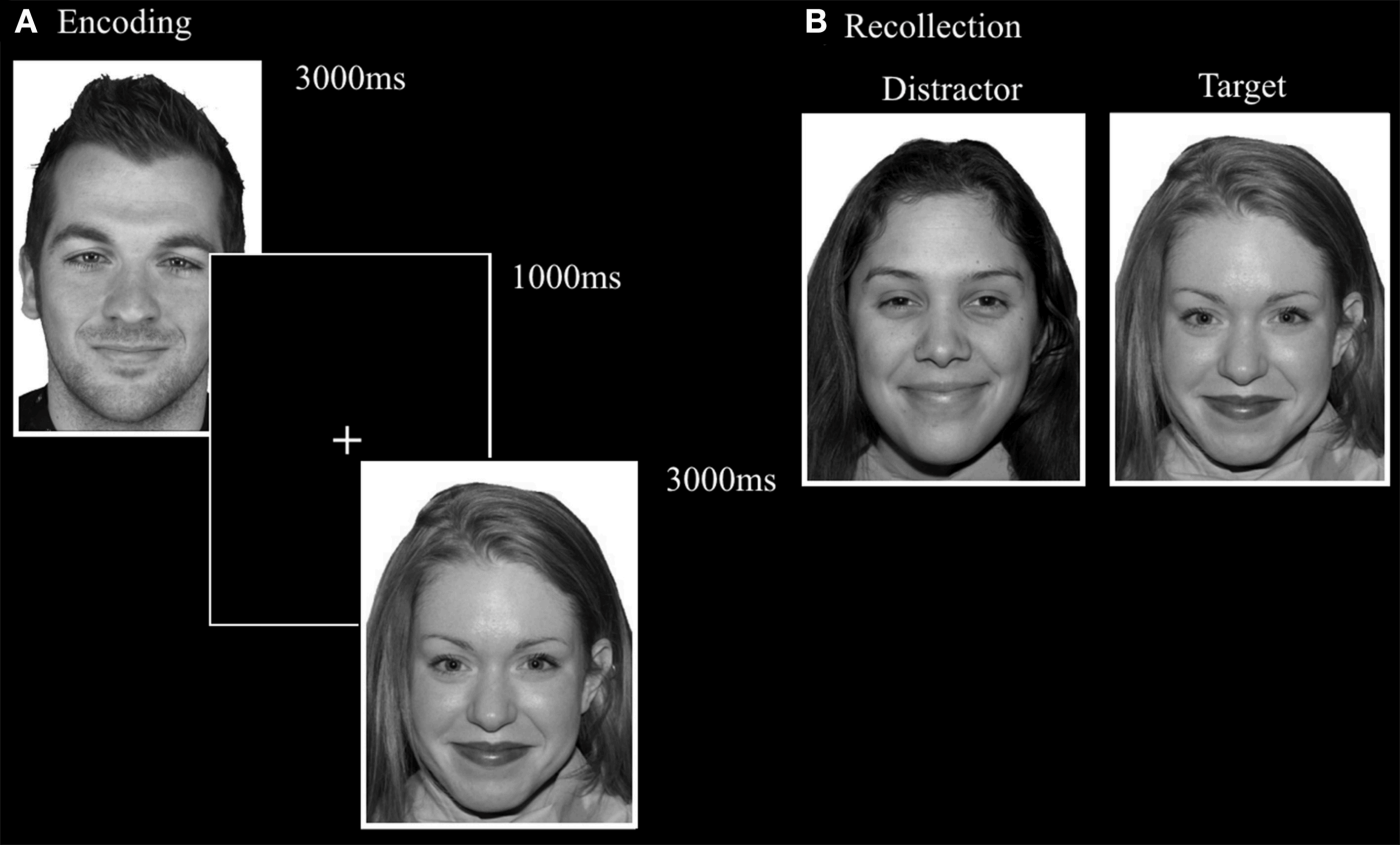
**Trial setup of a happy trial in the (A) encoding phase and (B) recollection phase of the FaMe-E**. Some identities are different from the actual stimuli due to copyright and permissions.

This task was designed by adapting the FaMe-N task by using stimuli containing emotional instead of neutral faces. Images were taken from the NimStim database (Tottenham et al., 2009) and stimuli created at Tilburg University. The stimuli consisted of 96 photographs (53 female) with direct eye gaze and frontal view. The individuals in the stimuli express fear, sadness, or happiness. There was no overlap in identities with the FaMe-N. The procedure was similar to the FaMe-N, but with 48 trials (16 per emotion) in both phases. The pictures making a stimulus pair were matched for emotion and hairstyle and in most trials also gender (see Figure 6).

### Analyses

Accuracies were calculated as the total proportion of correct responses for both the total score of each task and for each condition separately. Average response times from stimulus onset were calculated for the correct responses only. For all tasks, reaction times faster than 150 ms were excluded from analyses. In addition, for the identity matching task and part-to-whole matching task, reaction times longer than 3000 ms were excluded from analyses. For the other tasks, reaction times longer than 5000 ms were excluded from analyses. The number of outliers are reported in the results. One control subject did not complete the face and house part-to-whole matching task. The SPSS dataset can be downloaded through the supplementary materials.

In addition, the internal consistency was assessed with the Kuder Richardson coefficient of reliability (KR 20), reported as ρ_*KR*20_, which is analogous to Cronbach's alpha but suitable for dichotomous measures (Kuder and Richardson, 1937).

The results were analyzed using repeated measures GLMs, with the experimental factors as within subject variables and age group and gender as between subject variables. Interaction effects were further explored using *post-hoc* paired samples *t*-tests. The assumption of equality of error variances was checked with a Levene's test. The assumption of normality was not formally tested, as the sample is larger than 30 and repeated measures GLMs are quite robust against violations of normality.

Inversion scores were calculated by subtracting the accuracy and reaction time scores on the inverted presentation condition from the upright condition. A positive score indicates that accuracy was higher, or the reaction time was longer, on the upright condition. A negative score indicates higher accuracy or reaction times for the inverted condition. To assess whether a stronger configuration processing as measured by a higher accuracy inversion effect is related to improved face memory and emotion recognition, multiple linear regression analyses were performed with accuracy scores on the FaMe-N, FaMe-E, and both FEM tasks as dependent variable and age, gender, and four inversion scores (face identity, shoe identity, face-part, and house-part) as predictors. In addition, correlations between all tasks were calculated.

Lastly, percentile ranks of all tasks and correlations between all tasks were calculated and reported for both the accuracy scores and reaction times (see **Tables 8–11**).

## Results

### Face and shoe identity matching task

The task has a good internal consistency of ρ_*KR*20_ = 0.912. The following number of outliers were discarded; upright faces: a total of 0.86% outliers across ten participants (*M* = 3.2 trials, *SD* = 2.7, *min* = 1, *max* = 8); inverted faces: 0.7% across ten participants (*M* = 2.6 trials, *SD* = 2.7, *min* = 1, *max* = 10); upright shoes: 0.9% across 15 participants (*M* = 2.1 trials, *SD* = 2, *min* = 1, *max* = 7) and inverted shoes: 0.5% across four participants (*M* = 4.8 trials, *SD* = 5.7, *min* = 1, *max* = 13).

A repeated measures GLM on accuracy scores with category (faces, shoes) and orientation (upright, inverted) as within-subject factors and gender and age group as between-subject factors revealed a category by orientation interaction effect [*F*_(1, 54)_ = 16.955, *p* < 0.001, $\eta_{\text{p}}^{2}=0.24$]. Paired samples *t*-tests show that upright faces are recognized more accurately than inverted faces [*t*_(57)_ = 3.464, *p* = 0.001] and inverted shoes are recognized better than upright shoes [*t*_(57)_ = −2.254, *p* = 0.028]. Also, the middle aged group is less accurate overall [*F*_(1, 54)_ = 4.342, *p* = 0.042, $\eta_{\text{p}}^{2}=0.07$].

**Figure 7:**
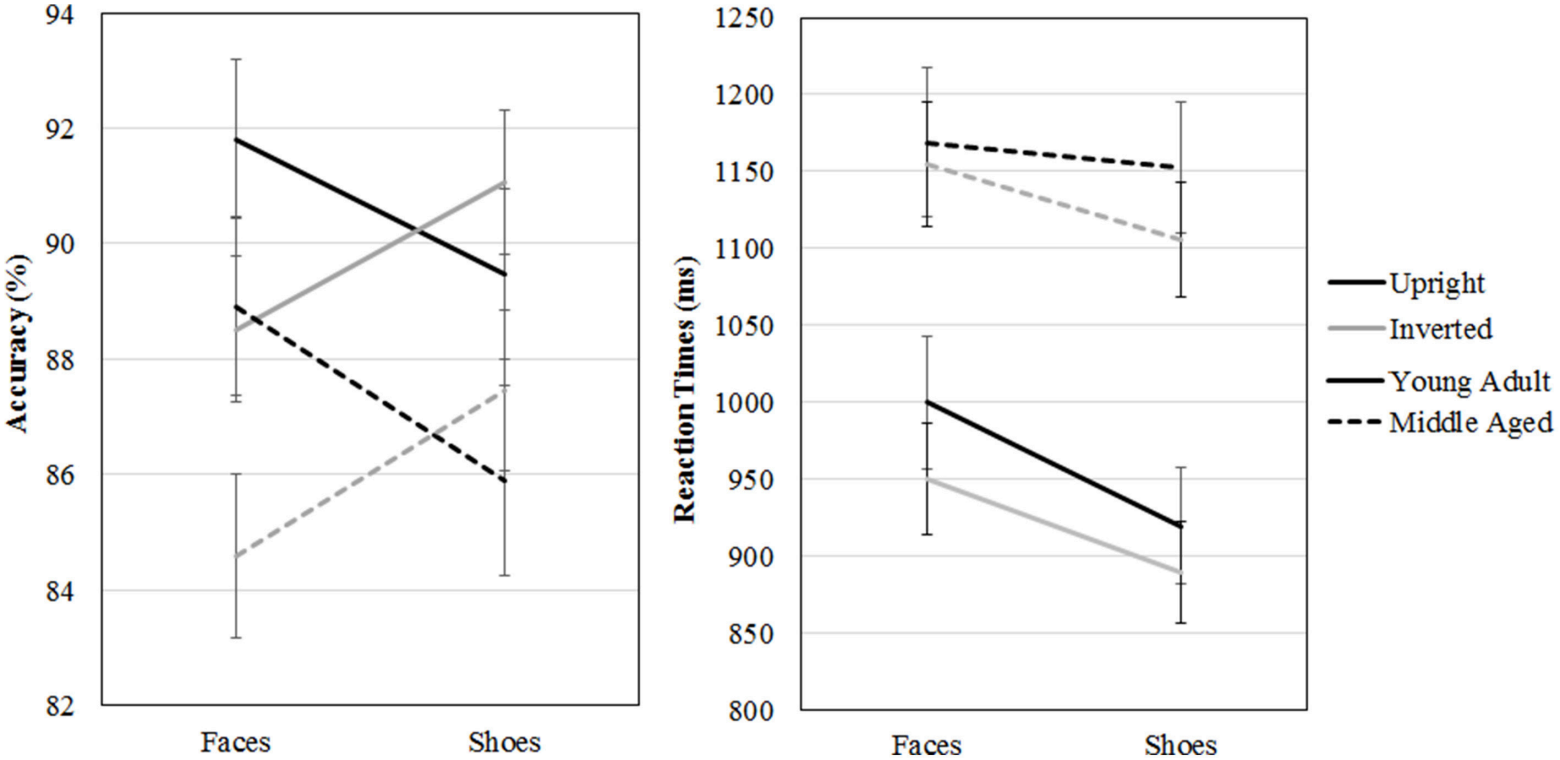
**Means and standard errors of the mean of the accuracy and reaction times on the face and shoe matching task, split by age group**.

A repeated measures GLM with a similar design on reaction times showed that faces are matched slower than shoes [*F*_(1, 54)_ = 16.063, *p* < 0.001, $\eta_{\text{p}}^{2}$ = 0.23], upright faces and shoes are matched slower than inverted ones [*F*_(1, 54)_ = 7.560, *p* = 0.008, $\eta_{\text{p}}^{2}$ = 0.12] and the middle aged group responded slower [*F*_(1, 54)_ = 15.174, *p* < 0.001, $\eta_{\text{p}}^{2}$ = 0.22; see Figure 7 and Table 1].

**Table 1:** **Means and standard deviations on the face and shoe matching task by age group**.

**Accuracy**		**Young adult**		**Middle aged**	
		***M* (%)**	***SD***	***M* (%)**	***SD***
Faces	Upright	92	7	89	9
	Inverted	89	8	85	8
Shoes	Upright	89	7	86	7
	Inverted	91	7	88	8
**Reaction times (ms)**		***M***	***SD***	***M***	***SD***
Faces	Upright	999	202	1162	280
	Inverted	951	202	1146	225
Shoes	Upright	920	175	1147	231
	Inverted	891	177	1100	201

### Face and house part-to-whole matching task

The task has a good internal consistency of ρ_*KR*20_ = 0.865. The following number of outliers were discarded; upright face parts: a total of 1.02% outliers across 38 participants (*M* = 2.7 trials, *SD* = 2.2, *min* = 1, *max* = 8); inverted face parts: 1.1% across 41 participants (*M* = 3.2 trials, *SD* = 3.2, *min* = 1, *max* = 13); upright house parts: 1.5% across 54 participants (*M* = 2.5 trials, *SD* = 2.8, *min* = 1, *max* = 12) and inverted house parts: 0.9% across 33 participants (*M* = 2.2 trials, *SD* = 1.6, *min* = 1, *max* = 6).

A repeated measures GLM on accuracy scores with category (faces, houses) and orientation (upright, inverted) as within-subject factors and gender and age group as between-subject factors revealed a three way age group by category by orientation interaction effect [*F*_(1, 53)_ = 5.413, *p* = 0.024, $\eta_{\text{p}}^{2}=0.09$]. Overall, both age groups are better at part to whole matching of houses [*F*_(1, 53)_ = 153.660, *p* < 0.001, $\eta_{\text{p}}^{2}=0.75$]. However, the young adult group is more accurately able to part to whole match upright than inverted faces [*t*_(31)_ = 5.369, *p* < 0.001], whereas the middle aged group is not [*t*_(24)_ = 0.952, *p* = 0.351], but no such group differences are found for house inversion [young adult group: *t*_(31)_ = −0.958, *p* = 0.345, middle aged group: *t*_(24)_ = −0.490, *p* = 0.628].

**Figure 8:**
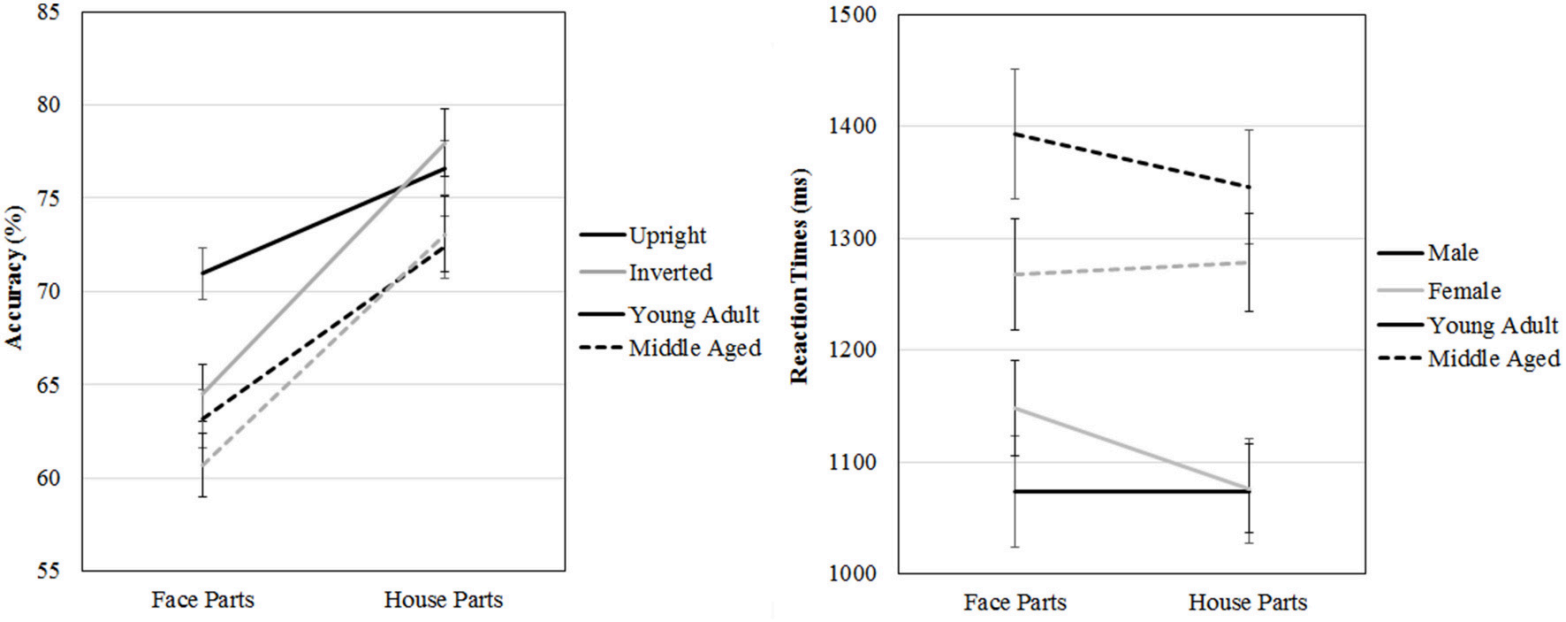
**Means and standard errors of the mean of the accuracy and reaction times on the face and house part-to-whole matching task split by age group**.

The same repeated measures GLM on reaction times revealed a three way gender by age group by category interaction effect [*F*_(1, 53)_ = 5.539, *p* = 0.022, η^2^p = 0.10]. To assess this effect, the repeated measures GLM with category (faces, houses) and orientation (upright, inverted) as within-subject factors and age group as between-subject factors was run for males and females separately. For the female group, a category by age group interaction effect is found [*F*_(1, 29)_ = 7.022, *p* = 0.013, $\eta_{\text{p}}^{2}=0.20$], whereas no significant effects were found for men (see Figure 8 and Table 2).

**Table 2:** **Means and standard deviations on the face and house part-to-whole matching task by age group**.

**Accuracy**		**Young adult**		**Middle aged**	
		***M* (%)**	***SD***	***M* (%)**	***SD***
Face parts	Upright	71	8	63	7
	Inverted	65	9	61	7
House parts	Upright	77	8	72	9
	Inverted	78	11	73	9
**Reaction times (ms)**		***M***	***SD***	***M***	***SD***
Face parts	Upright	1127	186	1346	218
	Inverted	1099	222	1299	215
House parts	Upright	1104	172	1307	163
	Inverted	1046	166	1309	178

### Facial expression matching task

#### Human facial expressions (FEM-H)

The task has a reasonably good internal consistency of ρ_*KR*__20_ = 0.769. The following number of outliers were discarded from 47 participants; 14% in total (Anger: 2.5%, disgust: 1.8%, fear: 3.4%, happy: 0.7%, sad: 3.5%, surprise: 2.2%, *M* = 10.4 trials, *SD* = 6.6, *min* = 1, *max* = 27).

A repeated measures GLM on the accuracy scores with emotion (fear, sadness, anger, disgust, surprise, and happy) as within subject variables and gender and age group as between subject variables showed a main effect of emotion [*F*_(5, 50)_ = 88.169, *p* < 0.001, $\eta_{\text{p}}^{2}=0.90$]. *Post-hoc* contrasts reveal that fear is recognized least accurate, worse than sadness [*F*_(1, 54)_ = 15.998, *p* < 0.001, $\eta_{\text{p}}^{2}=$ 0.23], on which accuracy rates are in turn lower than anger [*F*_(1, 54)_ = 63.817, *p* < 0.001, $\eta_{\text{p}}^{2}=$ 0.54]. Also, happy is recognized best with higher accuracy scores than surprise [*F*_(1, 54)_ = 49.157, *p* < 0.001, $\eta_{\text{p}}^{2}=$ 0.48].

**Figure 9:**
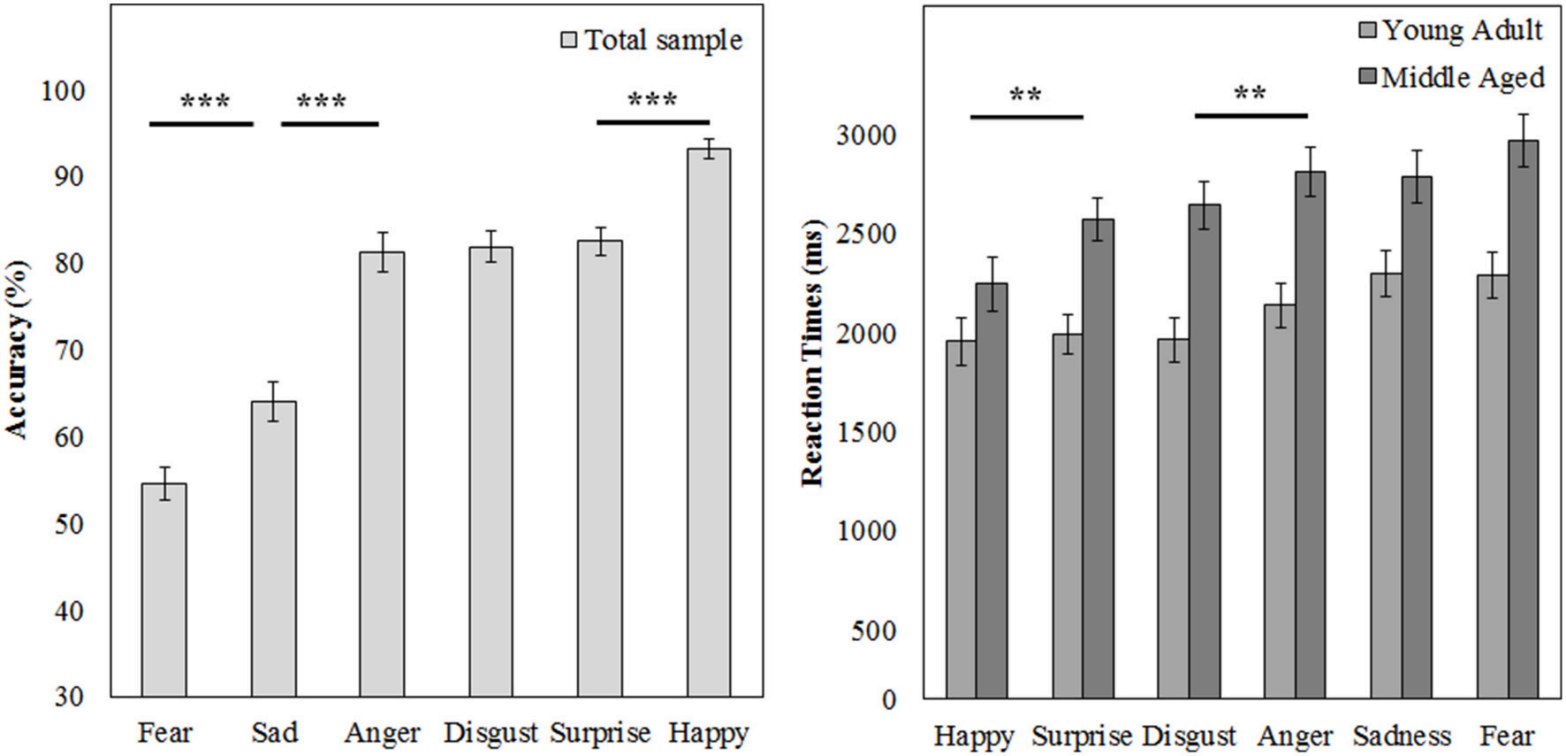
**Means and standard errors of the mean of the accuracy of the whole group and reaction times on the FEM-H split by age group**. ^***^p < 0.001, ^**^p < 0.05.

The same repeated measures GLM on the reaction time data revealed a main effect of emotion [*F*_(5, 50)_ = 15.055, *p* < 0.001, $\eta_{\text{p}}^{2}=$ 0.60]. Happy was also recognized fastest (as compared to surprise, *F*_(1, 54)_ = 7.873, *p* = 0.007, $\eta_{\text{p}}^{2}=$ 0.13] and disgust was recognized slower than anger [*F*_(1, 54)_ = 7.776, *p* = 0.007, $\eta_{\text{p}}^{2}=$ 0.13]. Also, the middle aged age group is slower overall [*F*_(1, 54)_ = 15.280, *p* < 0.001, $\eta_{\text{p}}^{2}=$ 0.22; see Figure 9 and Table 3].

**Table 3:** **Means and standard deviations on the FEM-H by age group**.

**Accuracy**	**Young adult**		**Middle aged**	
	***M* (%)**	***SD***	***M* (%)**	***SD***
Total	79	9	74	10
Anger	85	16	77	18
Fear	57	14	53	15
Happy	94	8	92	10
Disgust	82	13	82	12
Sad	69	17	59	15
Surprise	86	11	79	14
**Reaction times (ms)**	***M***	***SD***	***M***	***SD***
Total	2064	583	2628	493
Anger	2122	707	2819	541
Fear	2279	674	2976	662
Happy	1941	727	2253	647
Disgust	1951	627	2635	604
Sad	2276	733	2776	586
Surprise	1976	551	2574	598

#### Canine facial expressions (FEM-C)

The task has a good internal consistency of ρ_*KR*20_ = 0.847. From 35 participants, 5.3% of the trials were discarded (Anger: 1.1%, fear: 2.8%, happy: 1.4%, *M* = 6.3 trials, *SD* = 4.9, *min* = 1, *max* = 22).

A repeated measures GLM on the accuracy scores with emotion (fear, anger, and happy) as within subject variables and gender and age group as between subject variables revealed a main effect of emotion [*F*_(2, 53)_ = 37.049, *p* < 0.001, $\eta_{\text{p}}^{2}=$ 0.58]. Fear was recognized least accurately [as compared to happy, *F*_(1, 54)_ = 65.310, *p* < 0.001, $\eta_{\text{p}}^{2}=$ 0.55]. Also, the middle aged group was less accurate at this task than the young adult group [*F*_(1, 54)_ = 8.045, *p* = 0.006, $\eta_{\text{p}}^{2}=$ 0.13].

**Figure 10:**
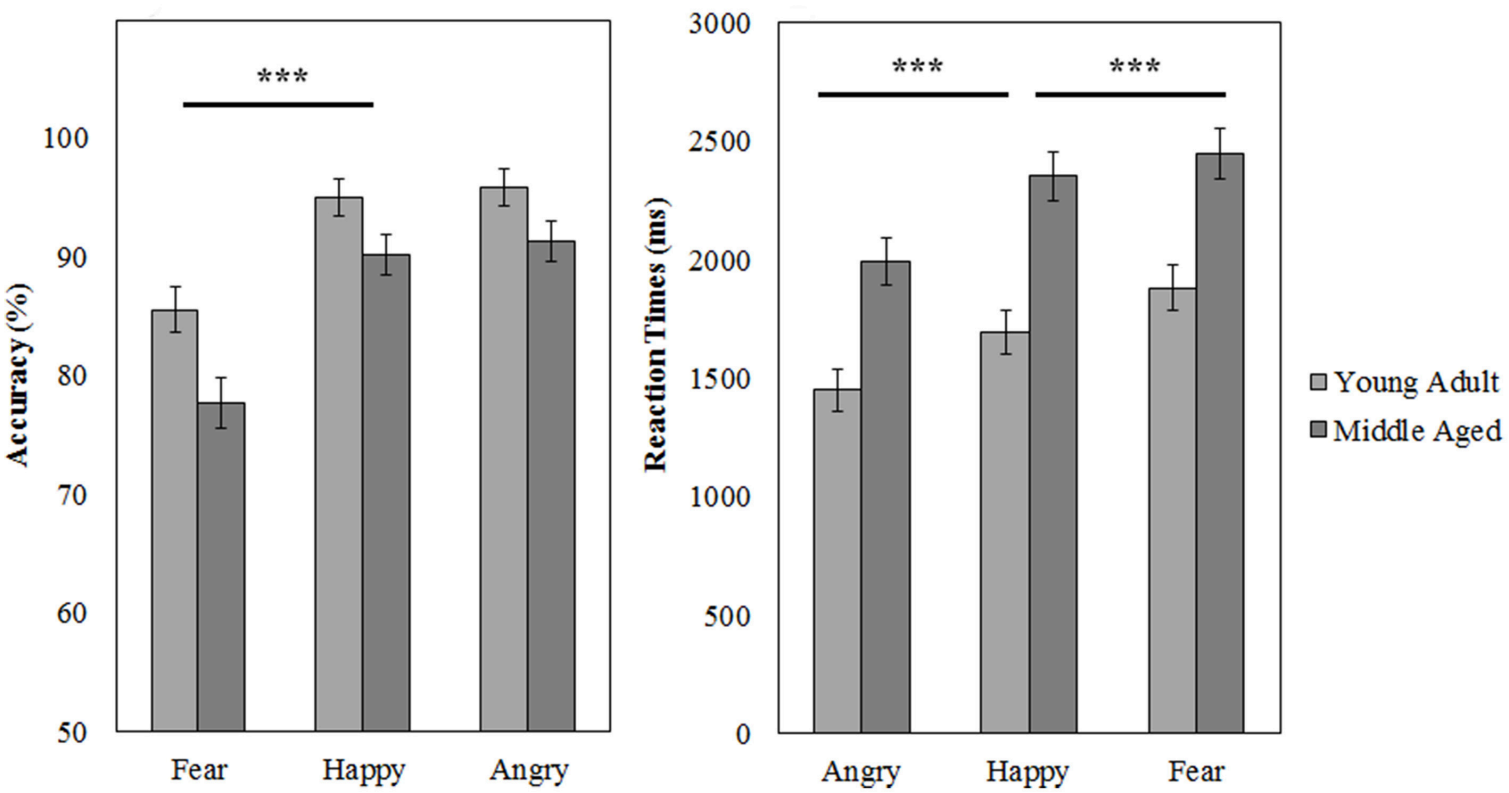
**Means and standard errors of the mean of the accuracy and reaction times on the FEM-Canine split by age group**. ^***^p < 0.001.

Similarly, for reaction times a main effect of emotion [*F*_(2, 53)_ = 66.335, *p* < 0.001, $\eta_{\text{p}}^{2}=$ 0.72] was observed; anger is recognized quicker than happy [*F*_(1, 54)_ = 74.880, *p* < 0.001, $\eta_{\text{p}}^{2}=$ 0.58], which is in turn recognized a faster than fear [*F*_(1, 54)_ = 17.588, *p* < 0.001, $\eta_{\text{p}}^{2}=$ 0.25]. Additionally, again the middle aged group is slower overall [*F*_(1, 54)_ = 19.817, *p* < 0.001, $\eta_{\text{p}}^{2}=$ 0.27; see Figure 10 and Table 4].

**Table 4:** **Means and standard deviations on the FEM-C by age group**.

**Accuracy**	**Young adult**		**Middle aged**	
	***M* (%)**	***SD***	***M* (%)**	***SD***
Total	92	8	86	7
Anger	96	8	91	9
Happy	86	11	77	11
Fear	95	9	90	9
**Reaction times (ms)**	***M***	***SD***	***M***	***SD***
Total	2064	583	2628	493
Anger	1446	532	1998	440
Happy	1874	613	2455	392
Fear	1683	571	2351	465

### Neutral face memory task (FaMe-N)

The task has a good internal consistency of ρ_*KR*20_ = 0.808. In total 232 trials (8%) were outliers across 50 participants (*M* = 4.6, *SD* = 4.5, *min* = 1, *max* = 24).

The participants scored on average 78% correct (*SD* = 12%) on the FaMe-N. No differences in accuracy scores on the FaMe-N are found for gender [*F*_(1, 54)_ = 0.238, *p* = 0.628, $\eta_{\text{p}}^{2}=$ 0.004] or age group [*F*_(1, 54)_ = 0.469, *p* = 0.496, $\eta_{\text{p}}^{2}=$ 0.009], nor is there any interaction effect.

**Figure 11:**
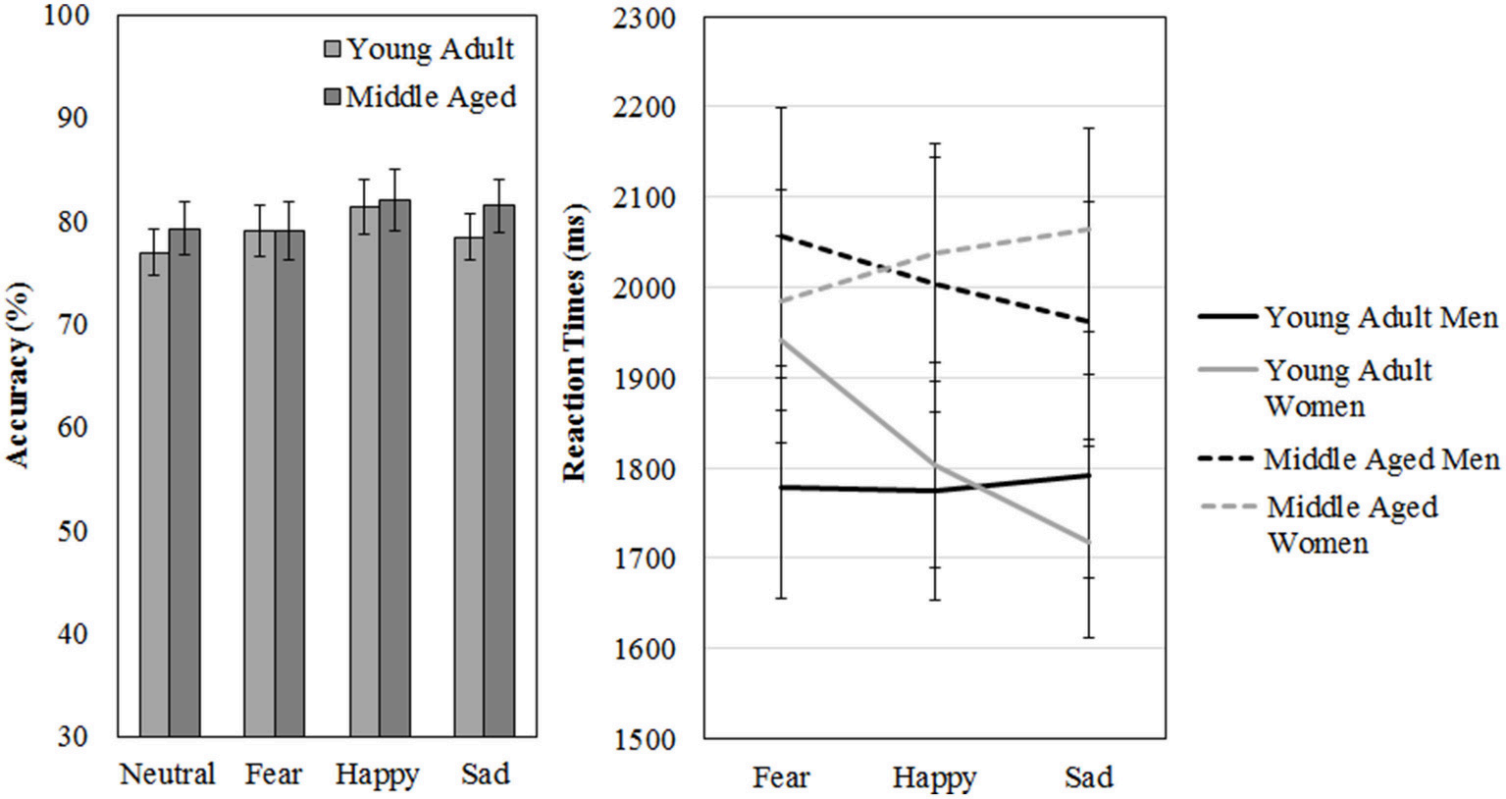
**Means and standard errors of the mean of the accuracy and reaction times on the FaMe-N and FaMe-E**.

Also, the average reaction time was 2121 ms (*SD* = 501) no difference in reaction times were found for gender [*F*_(1, 54)_ = 0.211, *p* = 0.648, $\eta_{\text{p}}^{2}=$ 0.004] but the effect of age group was near significance [*F*_(1, 54)_ = 3.768, *p* = 0.057, $\eta_{\text{p}}^{2}=$ 0.065; see Figure 11 and Table 5].

**Table 5:** **Means and standard deviations on the FaMe-N and the FaMe-E by gender and age group**.

**Accuracy (%)**		**Male**				**Female**			
		**Young adult**		**Middle aged**		**Young adult**		**Middle aged**	
		***M* (%)**	***SD***	***M* (%)**	***SD***	***M* (%)**	***SD***	***M* (%)**	***SD***
FaMe-Neutral	Total	77	16	81	11	77	13	78	10
FaMe-Emotion	Total	78	14	84	7	81	11	78	15
	Fear	78	13	82	7	80	16	76	17
	Happy	80	19	86	10	83	11	78	17
	Sad	77	15	83	7	80	12	80	13
**Reaction times (ms)**		***M***	***SD***	***M***	***SD***	***M***	***SD***	***M***	***SD***
FaMe-Neutral	Total	1920	532	2285	540	2090	483	2236	429
FaMe-Emotion	Total	1785	525	2007	246	1821	416	2025	430
	Fear	1778	544	2056	320	1942	540	1986	402
	Happy	1775	564	2003	275	1803	399	2038	540
	Sad	1791	514	1962	258	1718	414	2063	479

### Emotional face memory task (FaMe-E)

The task has a good internal consistency of ρ_*KR*20_ = 0.799. In total 125 trials (4.5%) were outliers across 34 participants (*M* = 3.7, *SD* = 3.5, *min* = 1, *max* = 19).

A repeated measures GLM on accuracy scores and reaction times scores with emotion (fear, happy, sad) as within-subject factors and gender and age group as between subject variables revealed no significant effects.

However, a gender by age group by emotion three-way interaction effect was found for reaction times, [*F*_(2, 53)_ = 3.197, *p* = 0.049, $\eta_{\text{p}}^{2}=$ 0.11]. Figure 11 shows that the pattern of results between men and women is reversed when the age groups are compared. It looks like young adult women seem quicker to recognize sadness than middle aged women: indeed, if the repeated measures is run for men and women separately, with emotion as within subject variables and age group as between, no effects of emotion or age group are found for men. However, for women, an emotion by age group interaction trend is found [*F*_(2, 29)_ = 2.987, *p* = 0.066, $\eta_{\text{p}}^{2}=$ 0.17; see Figure 11 and Table 5].

In addition, we directly compared the FaMe-N and FaMe-E using a repeated measures GLM on accuracy scores and reaction times scores on the neutral, fearful, happy, and sad conditions as within-subject factors and gender and age group as between subject variables, but no significant effects were found.

### Relationships between tasks

In the current sample, no significant predictive relationship between configuration processing as measured by the inversion effect and face memory scores were found (see Table 6).

**Table 6:** **Regression coefficients of the inversion scores on the tasks for configural and feature-based processing on the total scores of the Face Memory–Neutral and the Face Memory–Emotion task**.

**Step 1**	**FaMe-N**			**FaMe-E**		
	**B**	**Se B**	**β**	**B**	**Se B**	**β**
Constant	0.730	0.047		0.754	0.043	
Gender	−0.010	0.032	−0.039	0.003	0.029	0.013
Age	0.002	0.001	0.186	0.001	0.001	0.183
R^2^	0.036			0.034		
**Step 2**	**B**	**Se B**	**β**	**B**	**Se B**	**β**
Constant	0.728	0.059		0.778	0.052	
Gender	−0.010	0.036	−0.040	−0.008	0.032	−0.037
Age	0.001	0.001	0.182	0.001	0.001	0.150
Face inversion	0.001	0.003	0.034	−0.001	0.003	−0.066
Shoe inversion	−0.001	0.005	−0.027	−0.0004	0.004	−0.014
Face part inversion	0.000	0.003	−0.001	−0.003	0.003	−0.142
House part inversion	−0.001	0.003	−0.053	−0.003	0.003	−0.153
R^2^ change	0.004			0.044		

Similarly, no significant relationship between configuration processing and emotion recognition scores were found, aside from a negative effect of age on accuracy on the FEM-H and FEM-C, see Table 7. In addition, see Tables 8, 9 for correlations between the all the tasks and subtasks of the FEAST.

**Table 7:** **Regression coefficients of the inversion scores on the tasks for configural and feature-based processing on the total scores of the Facial Expression Matching- Human and Canine task**.

**Step 1**	**FEM-H**			**FEM-C**		
	**B**	**Se B**	**β**	**B**	**Se B**	**β**
Constant	0.831	0.034		0.955	0.028	
Gender	−0.003	0.023	−0.014	−0.011	0.020	−0.076
Age	−0.002	0.001	−0.264^*^	−0.001	0.001	−0.261
R^2^	0.07			0.034		
**Step 2**	**B**	**Se B**	**β**	**B**	**Se B**	**B**
Constant	0.829	0.041		0.965	0.035	
Gender	−0.003	0.026	−0.019	−0.019	0.021	−0.127
Age	−0.002	0.001	−0.255	−0.002	0.001	−0.319^*^
Face inversion	0.000	0.002	−0.024	0.001	0.002	0.091
Shoe inversion	0.000	0.003	0.017	−0.004	0.003	−0.181
Face part inversion	0.000	0.002	−0.021	−0.001	0.002	−0.092
House part inversion	−0.004	0.002	−0.227	0.000	0.002	−0.033
R^2^ change	0.054			0.044		

* *p < 0.05*.

**Table 8:** **Percentile ranks corresponding to accuracy scores (as percentage correct) split by age group for all tasks and subtasks**.

		**2**	**5**	**10**	**25**	**50**	**75**	**90**	**95**
**PERCENTILE RANKS YOUNG ADULT GROUP**									
FaMe-N		36	43	61	72	78	86	94	99
FaMe-E		48	55	65	71	79	91	96	98
Faces	Upr	72	76	80	89	93	98	98	98
	Inv	69	69	73	85	91	95	98	99
Shoes	Upr	64	71	83	86	91	94	97	98
	Inv	73	78	81	88	92	97	98	100
Face parts	Upr	50	54	60	66	71	78	81	86
	Inv	48	50	52	58	65	71	78	81
House parts	Upr	59	60	65	72	78	81	88	91
	Inv	41	54	65	72	78	84	91	93
FEM-H		53	60	65	74	82	85	90	90
FEM-C		57	75	85	88	94	97	98	99
**PERCENTILE RANKS MIDDLE AGED GROUP**									
FaMe-N		56	57	64	72	81	88	93	94
FaMe-E		42	47	65	77	82	90	94	96
Faces	Upr	69	70	75	82	91	95	98	99
	Inv	63	65	72	81	86	89	93	97
Shoes	Upr	69	70	75	81	88	91	94	96
	Inv	67	68	75	86	89	93	96	99
Face parts	Upr	48	49	53	57	64	67	73	75
	Inv	44	44	50	57	61	68	70	73
House parts	Upr	53	53	58	67	75	78	83	89
	Inv	55	57	62	66	72	79	87	90
FEM-H		50	54	63	67	73	83	87	91
FEM-C		67	70	75	83	88	92	94	97

**Table 9:** **Percentile ranks corresponding to reaction times split by age group for all tasks and subtasks**.

		**2**	**5**	**10**	**25**	**50**	**75**	**90**	**95**
**PERCENTILE RANKS THE YOUNG ADULT GROUP**									
FaMe-N		1220	1248	1329	1621	1996	2329	2589	3210
FaMe-E		869	978	1097	1462	1842	2145	2505	2582
Faces	Upr	671	693	733	832	974	1112	1293	1472
	Inv	670	673	708	782	908	1068	1235	1407
Shoes	Upr	591	663	707	777	922	1049	1204	1263
	Inv	605	617	666	741	879	1010	1177	1227
Face parts	Upr	591	718	910	1025	1130	1259	1281	1484
	Inv	481	544	909	997	1084	1230	1393	1499
House parts	Upr	688	774	882	1001	1073	1228	1332	1445
	Inv	577	710	921	954	1023	1161	1252	1361
FEM-H		1080	1090	1169	1659	2032	2482	2769	3267
FEM-C		798	887	1123	1256	1458	2048	2581	2911
**PERCENTILE RANKS FOR THE MIDDLE AGED GROUP**									
FaMe-N		1380	1389	1623	1948	2142	2631	2932	3194
FaMe-E		1359	1389	1466	1803	2025	2231	2510	2787
Faces	Upr	680	735	851	985	1114	1286	1560	1903
	Inv	683	713	846	988	1116	1328	1484	1503
Shoes	Upr	667	709	822	975	1134	1310	1483	1614
	Inv	722	746	815	935	1085	1280	1378	1391
Face parts	Upr	807	854	1026	1236	1353	1492	1648	1722
	Inv	720	783	980	1207	1319	1452	1621	1627
House parts	Upr	985	1011	1078	1190	1355	1401	1531	1599
	Inv	1017	1018	1074	1173	1274	1469	1555	1658
FEM-H		1885	1887	1915	2212	2642	3004	3264	3640
FEM-C		1687	1688	1699	1905	2245	2603	2738	2987

Furthermore, percentile ranks for accuracy scores as percentage correct and the reaction times are reported in Tables 8, 9, and the correlations between all tasks are reported in Tables 10, 11.

**Table 10:** **Correlation matrix between the accuracy scores on all tasks**.

		**FaMe-N**	**FaMe-E**	**Faces**		**Shoes**		**Face parts**		**House parts**		**FEM-H**
				**Upr**	**Inv**	**Upr**	**Inv**	**Upr**	**Inv**	**Upr**	**Inv**	
FaMe-E		0.67	–									
Faces	Upr	0.24	0.41	–	–	–	–	–	–	–	–	–
	Inv	0.15	0.36	0.51	–	–	–	–	–	–	–	–
Shoes	Upr	0.20	0.23	0.60	0.61	–	–	–	–	–	–	–
	Inv	0.27	0.34	0.60	0.63	0.69	–	–	–	–	–	–
Face parts	Upr	0.09	0.07	0.27	0.44	0.46	0.40	–	–	–	–	–
	Inv	0.15	0.25	0.46	0.47	0.48	0.56	0.50	–	–	–	–
House parts	Upr	0.03	0.06	0.44	0.49	0.44	0.44	0.50	0.47	–	–	–
	Inv	0.06	0.17	0.48	0.60	0.52	0.50	0.65	0.63	0.64	–	–
FEM-H		0.18	0.44	0.39	0.37	0.28	0.30	0.23	0.16	0.18	0.35	–
FEM-C		0.49	0.54	0.52	0.36	0.34	0.49	0.31	0.32	0.24	0.23	0.46

*White; p < 0.01, light gray; p < 0.05, dark gray; ns*.

**Table 11:** **Correlation matrix between the reaction times on all tasks**.

		**FaMe-N**	**FaMe-E**	**Faces**		**Shoes**		**Face parts**		**House parts**		**FEM-H**
				**Upr**	**Inv**	**Upr**	**Inv**	**Upr**	**Inv**	**Upr**	**Inv**	
FaMe-E		0.60	–	–	–	–	–	–	–	–	–	–
Faces	Upr	0.53	0.60	–	–	–	–	–	–	–	–	–
	Inv	0.50	0.57	0.86	–	–	–	–	–	–	–	–
Shoes	Upr	0.53	0.51	0.84	0.89	–	–	–	–	–	–	–
	Inv	0.46	0.54	0.77	0.91	0.89	–	–	–	–	–	–
Face parts	Upr	0.39	0.50	0.63	0.71	0.70	0.76	–	–	–	–	–
	Inv	0.45	0.44	0.52	0.63	0.61	0.66	0.78	–	–	–	–
House parts	Upr	0.42	0.54	0.68	0.74	0.74	0.77	0.85	0.74	–	–	–
	Inv	0.41	0.46	0.57	0.68	0.68	0.71	0.83	0.80	0.89	–	–
FEM-H		0.40	0.54	0.43	0.47	0.53	0.53	0.35	0.36	0.53	0.48	–
FEM-C		0.59	0.57	0.52	0.54	0.61	0.58	0.45	0.44	0.58	0.48	0.81

*All correlations are significant at the p < 0.01 level*.

## Discussion

In this study, we provide normative data of a large group of healthy controls on several face and object recognition tasks, face memory tasks and emotion recognition tasks. The effects of gender and age were also reported. All tasks have a good internal consistency and an acceptable number of outliers.

Firstly, face and object processing and configuration processing were assessed. As expected, upright face recognition is more accurate than inverted face recognition, in line with the face inversion effect literature (Yin, 1969; Farah et al., 1995). Interestingly, even though the middle aged group was less accurate than the young adults group, their response patterns regarding face and object inversion were comparable. As configurational processing measured by (upright-inverted) inversion scores was not influenced by gender or age, this is a stable effect in normal subjects. The absence of any interaction effects with age group or gender indicate that category specific configuration effects are stable across gender and between young adulthood and middle age. This implies it is a suitable index to evaluate in prosopagnosia assessment. Secondly, the face and house part to whole matching task seems to be a harder task than the whole face and shoe matching task, as indicated by overall lower accuracies. Young adults are more sensitive to inversion in this task.

Thirdly, we found that fear and sadness recognition on our FEM-H task was quite poor, but that anger, disgust, surprise and happiness were recognized above 80% accuracy. Similarly, canine emotions were recognized very well, although fear was also the worst recognized canine emotion and the older age group scored slightly worse and slower on this task, confirming that this subtest provides a good control.

Lastly, no effects of gender or age were found on neutral face memory, and participants scored quite well on the task, with an average of almost 80% correct. Similarly, no clear effects of age, gender or emotion were found on face memory as measured with the FaMe-E, except that it seems that middle aged women are slower to recognize previously seen identities when they expressed sadness. Interestingly, this is in line with the “age-related positivity effect” (Samanez-Larkin and Carstensen, 2011; Reed and Carstensen, 2012). In general, the results corroborate those from other studies on the effect of emotion on memory (Johansson et al., 2004), but a wide variety of results has been reported in the literature (Dobel et al., 2008; Langeslag et al., 2009; Bate et al., 2010; D'Argembeau and Van der Linden, 2011; Righi et al., 2012; Liu et al., 2014). In addition, we did not find any relationships between configuration perception and face memory. This can be due to the fact that unlike in samples with DPs and controls, there is less variability in inversion scores and memory scores (i.e., most participants will not have any configuration processing deficits similar to DPs and in contrast to DPs, most controls are not severely limited on face memory).

The results indicate that age is most likely a modulating factor when studying face and object processing, as the responses of the middle aged group is often slower. One explanation besides a general cognitive decline with age can be found in the literature on the effect of age on facial recognition, where an “own-age bias” is often found (Lamont et al., 2005; Firestone et al., 2007; He et al., 2011; Wiese, 2012). The “own-age bias” in face recognition refers to the notion that individuals are more accurate at recognizing faces from individuals belonging to the age category of the observer. For instance, children are better at recognizing child faces and adults are better at recognizing adult faces. Future researchers wishing to use the FEAST should compare the results of their participants with the appropriate age group, or should control for the effects of age or ideally, test age-matched controls. Gender on the other hand does not seem so influential, but this article provides guidelines and data for both gender and age groups regardless.

Some limitations of the FEAST should be noted. One is the lack of a non-face memory control condition using stimuli with comparable complexity. However, a recent study with a group of 16 DPs showed that only memory for faces, in contrast to hands, butterflies and chairs was impaired (Shah et al., 2014), so for this group this control condition might not be necessary. Also, the specific effects of all emotions, valence and arousal may be taken into account in future research. The face memory test could be complemented with the use of test images that show the face in the test phase from a different angle that in the training phase as is done in the matching tests. In addition, the low performance on fear recognition should be assessed. In short, the FEAST provides researchers with an extensive battery for neutral and emotional face memory, whole and part-to-whole face and object matching, configural processing and emotion recognition abilities.

## Author contributions

All authors contributed significantly to the concept and design of the work. EH collected and analyzed the data. All authors contributed to data interpretation. EH drafted the first version of the manuscript and BD and JV revised.

## Funding

National Initiative Brain & Cognition; Contract grant number: 056-22-011. EU project TANGO; Contract grant number: FP7-ICT-2007-0 FETOpen. European Research Council under the European Union's Seventh Framework Programme (ERC); Contract grant number: FP7/2007–2013, agreement number 295673. JV is a post-doctoral research fellow for FWO-Vlaanderen.

### Conflict of interest statement

The authors declare that the research was conducted in the absence of any commercial or financial relationships that could be construed as a potential conflict of interest.
